# Biochemical predictors of postoperative atrial fibrillation following cardiac surgery

**DOI:** 10.1186/s12872-021-01981-z

**Published:** 2021-04-09

**Authors:** Sevket T. Turkkolu, Emre Selçuk, Cengiz Köksal

**Affiliations:** Department of Cardiovascular Surgery, Faculty of Medicine, Bezmialem Vakif University, Adnan Menderes Bulvarı, Vatan Caddesi, Fatih/İstanbul, 34093 Istanbul, Turkey

**Keywords:** Postoperative atrial fibrillation, Cardiac surgery, Biochemical predictor, Clinical predictors, Treatment-resistant atrial fibrillation, Independent predictor

## Abstract

**Background:**

New-onset postoperative atrial fibrillation (POAF) is common after cardiac surgery. Early identification of its risk factors during the preoperative period would help in reducing the associated morbidity, mortality, and healthcare costs.

**Aim of the study:**

This study aimed to identify the predictors of POAF following open cardiac surgery, with emphasis on biochemical parameters.

**Methods:**

A total of 1191 patients with no preoperative atrial fibrillation (AF) and undergoing open cardiac surgery for any reason were included in this retrospective study. Data on clinical and biochemical parameters, the occurrence of new-onset AF, and its clinical course were retrieved from the hospital database.

**Results:**

During the early postoperative period 330 patients (27.7%) developed atrial fibrillation, at median third postoperative day (range 1–6 days) and 217 (65.8%) responded to treatment. Multivariate analysis identified the following as the significant independent predictors of any POAF: EF < 60% (Odds ratio (OR), 2.6), valvular intervention (OR, 2.4), liver failure (OR, 2.4), diabetes (OR, 1.6), low hematocrit (OR, 2.1), low thrombocyte (OR, 5.6), low LDL (OR, 1.6), high direct bilirubin (OR, 2.0), low GFR (OR, 1.6), and high CRP (OR, 2.0). Following parameters emerged as significant independent predictors of persistent AF: EF < 60% (OR, 1.9), diabetes (OR, 2.1), COPD (OR, 1.8), previous cardiac surgery (OR, 3.1), valvular intervention (OR, 2.4), low hematocrit (OR, 1.9), low LDL (OR, 2.1), high HbA1c (OR, 2.0), and high CRP (OR, 2.7).

**Conclusions:**

Certain parameters assessed during preoperative physical and laboratory examinations have the potential to be used as markers of POAF.

## Introduction

Although new-onset postoperative atrial fibrillation (POAF) is a complication that might occur following any type of surgery, it is more common after cardiac surgery. The proportion of patients suffering POAF may be as high as 64% following procedures for valvular pathology [1]. POAF is an important complication that may lead to hemodynamic instability, thromboembolism, transient ischemic attack, stroke, end-organ failure, prolonged hospital stay, increased mortality, and increased healthcare costs [2]. It is not only associated with increased postoperative mortality, but also with a significant decrease in long-term survival rates [3].

Until now, the exact pathophysiology of POAF following cardiac surgery has not been fully elucidated [4]. Potential predisposing factors that have been implicated include age, type of cardiac surgery, presence of myocardial ischemia, atrial distension, and pre-existing cardiac conditions [5, 6]. Other potential contributing conditions include those that cause sympathetic activation due to surgical stress, such as hypovolemia, intraoperative hypotension, anemia, and pain [7]. Hypervolemia may also cause atrial fibrillation through an increase in the atrial volume and altered cardiac conduction [7, 8]. Myocardial injury due to surgery and pericardial inflammation have also been implicated as potential pathogenetic mechanisms [9].

Unfortunately, no definitive preventive and therapeutic protocols have been developed due to poorunderstanding of the pathogenesis of POAF following cardiac surgery. It may be possible to reduce the high morbidity, mortality, and treatment costs associated with POAF by early identification of risk factors during the preoperative period.

Other triggering mechanisms for atrial fibrillation that occur after cardiac surgery may includeelectrophysiological, biochemical, and metabolic imbalances. In this regard, several previous studies have demonstrated associations between certain biochemical parameters and the development of POAF [4, 10–14]. It is crucial to examine comprehensive biochemical parameters and clinical features in a large patient population to identify the most important predictors for AF.

Thus, the objective of the current study was to identify biochemical and clinical predictors of postoperative new-onset atrial fibrillation in patients undergoing open cardiac surgery for any indication.

## Materials and methods

### Patients and data extraction

A total of 1191 patients with preoperative sinus rhythm who underwent cardiac surgery for any indication between January 2015 and December 2019 were included in our unit. Data on clinical and biochemical parameters, the occurrence of new-onset AF and its clinical course were retrieved from our hospital database and retrospectively evaluated. AF diagnosis was based on the criteria proposed by AHA/ACC/HRS 2014 Guidelines for Atrial Fibrillation [15]. AF was defined as the demonstration of AF for a minimum duration of 30 s using electrocardiography (ECG) recordings.

Blood samples were taken 48 h before surgery for all patients according to institutional protocol. Exclusion criteria were (1) non-sinus rhythm before surgery (2) history of paroxysmal or chronic AF (3) implanted cardiac devices (4) electrophysiologic ablation history. All postoperative ECG records were assessed by double-checked two independent investigators.

The study protocol was approved by the local ethics committee (Bezmialem Vakif University, Ethics Committee for Non-interventional Research; date, March 6, 2020; number 54022451–050.05.04) and the study was conducted in accordance with the Declaration of Helsinki and its later amendments.

### AF management

Primary objectives of the treatment of new-onset AF following open cardiac surgery included rate control, restoration of sinus rhythm, and anticoagulation. A beta-blocker or calcium canal blocker together with intravenous amiodarone followed by oral administration were used as the first-line management strategy for ventricular rate control and restoration of normal sinus rhythm. However, in hemodynamically unstable patients without contraindication to electrical cardioversion, initial electrical cardioversion was performed followed by the treatment described above. In patients unresponsive to amiodarone, oral propafenone or sotalol was used. In addition, all patients received warfarin to prevent the risk of stroke, with a target INR between 2.0–3.0 or 2.5–3.5, depending on the type of cardiac surgery. Following detection of AF, patients were hospitalized for a minimum duration of one week after starting the medical treatment. Restoration of normal sinus rhythm with a resting heart rate of < 80 bpm was considered to indicate successful treatment. Patients remaining unresponsive to these therapeutic measures were considered as having persistent AF and were discharged home with the most recent prescribed treatment. On the other hand, patients with the restoration of normal sinus rhythm were discharged with dose adjustment.

### Statistical analysis

IBM SPSS Statistics version 21.0 software (SPSS Inc., Chicago, IL) was used for the analysis of data. Descriptive data are presented in number (percentage), mean ± standard deviation, or median (range), where appropriate. Categorical variables were compared using Pearson’s chi-square test or Fisher’s exact test. Depending on the normality of the data, continuous variables were compared using Mann–Whitney U or student’s t test for independent samples. Kolmogorov–Smirnov test were used to test normality. Multivariate analysis using stepwise logistic regression (forward conditional) was done for the examination of significant independent predictors of any AF development and persistent AF development. Two-sided *p* values < 0.05 were considered indication of statistical significance.

## Results

Table 1 shows the demographical, clinical, and surgical characteristics of the patients. A total of 330 patients (27.7%) developed atrial fibrillation during the early postoperative period, at median third postoperative day (range 1–6 days). Among them, 217 (65.8%) responded to treatment, the remaining 113 (34.2%) had persistent AF.

**Table 1:** Demographical and clinical characteristics of the patients

	Overall	Patients without POAF	Patients with POAF	*p*
Number of patients	1191 (100)	861 (72.3)	330 (27.7)	–
*Demographics*				
Age (year), median (min–max)	63 (16–87)	61 (16–83)	69 (29–87)	< 0.001
Male gender, n (%)	691 (58.0%)	501 (58.1)	190 (56.8)	0.94
*Comorbidities n (%)*				
Diabetes	272 (22.8%)	177 (20.6)	95 (28.8)	0.002
Hypertension	652 (54.7%)	440 (51.1)	212 (64.2)	< 0.001
Chronic renal failure	152 (12.8%)	93 (10.8)	59 (17.9)	< 0.001
COPD	182 (15.3%)	113 (13.1)	69 (20.9)	< 0.001
Liver failure	24 (2.0%)	10 (1.2)	14 (4.2)	< 0.001
Cerebrovascular disease	47 (3.9%)	27 (3.1)	20 (6.1)	0.02
Peripheral artery disease	135 (11.3%)	89 (10.3)	46 (13.9)	0.08
Previous cardiac surgery	59 (5.0%)	31 (3.6)	28 (8.5)	< 0.001
*Perioperative factors*				
EF (%), median (min–max)	60 (30–65)	60 (30–60)	55 (36–65)	< 0.001
Type of operation, n (%)				
Isolated CABG	599 (50.3%)	477 (55.4)	122 (37.0)	< 0.001
Isolated valvular intervention	374 (31.4%)	241 (28.0)	133 (40.3)	< 0.001
CABG plus valvular intervention	150 (12.6%)	82 (9.5)	68 (20.6)	< 0.001
Other	68 (5.7%)	61 (7.1)	7 (2.1)	< 0.001
CPB time (min), median (min–max)	138 (49–319)	136 (49–319)	143 (58–270)	< 0.001
CC time (min), median (min–max)	70 (19–245)	69 (22–245)	75 (19–165)	< 0.001

Unless otherwise stated, data presented as *n* (%)

CABG, coronary artery bypass grafting; COPD, chronic obstructive pulmonary disease; SD, standard deviation, EF, ejection fraction; CC, cross-clamp

### Predictors of postoperative AF development

Patients who developed postoperative atrial fibrillation were significantly older, had lower ejection fraction (EF), and longer cardiopulmonary bypass and cross-clamp times. Postoperative atrial fibrillation was more common among patients that underwent valvular operations, concomitant CABG and valvular operations, and in patients with diabetes, hypertension, chronic renal failure, COPD, liver failure, previous cardiac surgery, cerebrovascular disease, and peripheral artery disease Multivariate analysis identified the following clinical/surgical parameters as significant independent predictors of postoperative AF development: EF < 60% (Odds ratio (OR), 2.6), valvular intervention (OR, 2.4), and diabetes (OR, 1.6).

Among hematological/biochemical parameters, low hemoglobin (< 12.5 g/dL), low hematocrit (< 35%), low thrombocyte count (< 142 × 10^9^/L), low calcium (< 8.4 mg/dL), low glomerular filtration rate (≤ 90 mL/min/1.73m^2^), low HDL (< 35 mg/dL), low LDL (< 100 mg/dL), high HbA1c (> 6.5%), high AST (> 35 U/L), high ALT (> 55 U/L),high total bilirubin (> 1.2 mg/dL),high direct bilirubin (> 0.5 mg/dL), high insulin (> 23.4 mIU/L), high glucose (> 105 mg/dL), high creatinine (> 1.25 mg/dL), high blood urinary nitrogen (> 25.7 mg/dL), high CRP (> 5 mg/L), high urea levesl (> 43 mg/dL) were associated with increased frequency of postoperative AF development on univariate analysis (*p* < 0.05 for all comparisons). Multivariate analysis identified the following hematological/biochemical parameters as significant independent predictors of postoperative AF development: low hematocrit (OR, 2.1), low thrombocyte count (OR, 5.6), low LDL (OR, 1.6), high direct bilirubin (OR, 2.0), low GFR (OR, 1.6), and high CRP (OR, 2.0). Table 2 shows the details of significant independent clinical/surgical and hematological/biochemical predictors of early postoperative AF development on multivariate analysis.

**Table 2:** Significant independent predictors of early postoperative AF development on multivariate analysis

Predictors	Predictors for any AF*	Predictors for persistent AF*
*Clinical/surgical predictors*		
Low EF (< 60%)	2.6, 95% CI 2.0–3.4, *p* < 0.001	1.9, 95% CI 1.2–2.9, *p* = 0.008
Valvular intervention	2.4, 95% CI 1.8–3.2, *p* < 0.001	2.4, 95% CI 1.6–3.8, *p* < 0.001
Diabetes	1.6, 95% CI 1.2–2.2, *p* = 0.004	2.1, 95% CI 1.3–3.3, *p* = 0.002
COPD		1.8, 95% CI 1.1–2.9, *p* = 0.018
Previous cardiac surgery		3.1, 95% CI 1.7–5.8, *p* < 0.001
*Hematological/biochemical predictors*		
Low hematocrit (< 35%)	2.1, 95% CI 1.5–2.9, *p* < 0.001	1.9, 95% CI 1.2–2.9, *p* = 0.006
Low thrombocyte (< 142 × 10^9^/L)	5.6, 95% CI 1.1–29.3, *p* = 0.042	
Low LDL (< 100 mg/dL)	1.6, 95% CI 1.2–2.1, *p* = 0.004	2.1, 95% CI 1.3–3.1, *p* = 0.001
High direct bilirubin (> 0.5 mg/dL)	2.0, 95% CI 1.4–2.9, *p* < 0.001	
Low GFR (≤ 90 mL/min/1.73m^2^)	1.6, 95% CI 1.1–2.4, *p* = 0.026	
High CRP (> 5 mg/L)	2.0, 95% CI 1.2–3.2, *p* = 0.004	2.7, 95% CI 1.5–4.7, *p* = 0.001
High HbA1c (> 6.5%)		2.0, 95% CI 1.3–3.1, *p* = 0.001

AF, atrial fibrillation; COPD, chronic obstructive pulmonary disease; CRP, C-reactive protein; EF, ejection fraction; GFR, glomerular filtration rate; LDL, low-density lipoprotein

*Odds ratio, 95% confidence interval, *p* value

### Predictors of early postoperative persistent AF development

Patients who developed postoperative atrial fibrillation were significantly older (median 66 vs. 62 years, *p* < 0.001), had lower ejection fraction (median 55% vs. 60%, *p* < 0.001), and longer cardiopulmonary bypass (median 153 vs. 140 min, *p* = 0.001) and cross-clamp times (median 84 vs.75 min, *p* < 0.001). Persistent postoperative atrial fibrillation was more common among patients that underwent valvular operations, and in patients with diabetes, hypertension, COPD, liver failure, previous cardiac surgery, cerebrovascular disease, and peripheral artery disease (*p* < 0.05 for all). Multivariate analysis identified the following clinical/surgical parameters as significant independent predictors of persistent postoperative AF development: EF < 60% (OR, 1.9), diabetes (OR, 2.1), COPD (OR, 1.8), previous cardiac surgery (OR, 3.1), and valvular intervention (OR, 2.4).

Among hematological/biochemical parameters, low hemoglobin (< 12.5 g/dL), low hematocrit (< 35%), low calcium (< 8.4 mg/dL), low sodium (< 135 mEq/L), low glomerular filtration rate (≤ 90 mL/min/1.73m^2^), low HDL (< 35 mg/dL), low LDL (< 100 mg/dL), low insulin (< 2.4 mIU/L), high HbA1c (> 6.5%), high ALT (> 55 U/L), high direct bilirubin (> 0.5 mg/dL), high glucose (> 105 mg/dL), high creatinine (> 1.25 mg/dL), high blood urinary nitrogen (> 25.7 mg/dL), high C-reactive protein (> 5 mg/dL), and high urea levels (> 43 mg/dL) were associated with increased frequency of persistent postoperative AF development on univariate analysis (*p* < 0.05 for all comparisons). Multivariate analysis identified the following hematological/biochemical parameters as significant independent predictors of persistent postoperative AF development: low hematocrit (OR, 1.9), low LDL (OR, 2.1), high HbA1c (OR, 2.0), and high CRP levels (OR, 2.7). Table 2 shows the details of significant independent clinical/surgical and hematological/biochemical predictors of persistent AF development on multivariate analysis.

## Discussion

The results of our study showed that persistent or non-persistent atrial fibrillation developing early after open cardiac surgery is influenced not only by clinical factors but also by several biochemical parameters, some of which are modifiable. In order to contribute to the current knowledge, a wide range of patients and several biochemical parameters were investigated, considering that possibly POAF is a multifactorial condition with an interplay between many modifiable or non-modifiable clinical, biochemical, and hematologic factors.

Some clinical variables such as left ventricular dysfunction, liver failure, diabetes, COPD ve previous cardiac surgery which were also emphasized in previous studies, were found to be an important risk factor for POAF or persistent POAF in our study population [3, 16–19]. In this regard, our study confirms the importance of these clinical risk factors for PAOF, which were previously investigated in different studies. In addition, these findings indirectly indicate that the clinical features of our study population are similar to other studies and reflect the current scenario of cardiac surgery patients.

As suggested by many previous studies, POAF may arise due to a variety of mechanisms triggered by different events. Furthermore, the risk of POAF differs significantly between varying types of surgery, due to the inherent nature of open cardiac procedures [20]. This latter point should definitely be taken into consideration in studies aiming to determine marker(s) for the risk of POAF, and a comprehensive panel of biochemical and hematologic parameters should be evaluated individually or combined, in a wide spectrum of patients undergoing different types of surgery, to increase the reliability of study findings.

CRP represents the most extensively studied marker for POAF risk, regardless of the type of surgery. Almost all previous studies and meta-analyses uniformly indicate that elevated CRP is closely associated with the risk of POAF, and our results are consistent with such findings [3, 21]. Preoperative high CRP levels possibly reflect a high basal level of systemic inflammation [22, 23].

A recent meta-analysis examining predictive hematological parameters for new-onset AF revealed that patients with AFhad significantly lower mean platelet count than patients without AF [24]. In another meta-analysis focusing on hematological markers for AF after cardiac surgery, the correlation between low platelet count and increased frequency of POAF was confirmed [25]. This inverse correlation also persisted in our study. Another hematological parameter in our study, anemia, has not yet been universally emphasized as a predictor for POAF [25]. It is important to urgently confirm this finding in external populations because anemia, which is also associated with persistent POAF in our study, is shown to be a risk factor for long-term mortality in patients with nonvalvular AF [26]. Thrombocytopenia and anemia are possibly reflect the presence of comorbidities such as chronic kidney disease, underlying cancer, and liver damage which may be associated with POAF. In addition, these two parameters may be associated with postoperative increased blood product use, which has been shown as a risk factor for POAF [27].

Although the association between diabetes and POAF has been previously examined, the role of HbA1c, the main marker of poor glycemic control, in In one of the few reported studies, contrary to our observations, it has been found that HbA1c was not an independent preoperative predictor of POAF [28]. Conversely, similar to our findings, others reported that HbA1c may represent a highly significant predictor of POAF, not only in patients with known diabetes but also in diabetes-free patients as well as subjects with undiagnosed diabetes [29, 30]. These results indicate the importance of checking perioperative blood glucose regulation in all patients undergoing cardiac surgery, regardless of the known diabetes history.

One of the most interesting results of this study is the inverse relationship between preoperative LDL levels and the risk of POAF. The relationship between hypolipoproteinemia and the increased frequency of AF has been repeatedly emphasized in previous cornerstone studies [31–33]. The inverse relationship between age and hyperthyroidism, which are risk factors for AF, and lipid levels, and the membrane-stabilizing and anti-inflammatory effect of lipoproteins are considered as possible explanatory mechanisms [34]. However, the relationship between POAF with lipid levels has not been extensively studied. Contrary to our study, a recent retrospective study with a relatively small cohort (A total of 100 CABG patients) found a positive correlation between preoperative high LDL level and the incidence of postoperative AF [35]. The relationship between preoperative statin use and POAF has been studied more than in lipid profile. While a significant portion of the studies revealed that the preoperative use of statins reduced the risk of POAF due to the pleiotropic effect, some studies showed that there was no significant reduction in the risk of POAF with preoperative statin use [36, 37]. Based on the results of statin studies, it is not possible to make inferences regarding the relationship between lipid profile and POAF. Studies in different populations are urgently needed to resolve dilemmas on this issue and to elucidate the mechanisms underlying our findings.

The relationship between AF and bilirubin levels remains controversial. Demir et al. reported that there is an inverse relationship between serum bilirubin levels and nonvalvular AF [38]. However, growing evidence suggests that higher bilirubin levels may be associated with AF [39–41]. We did not come across any other study that indicates high bilirubin levels as a predictor of POAF. We determined that the high level of direct bilirubin is an important predictor for POAF. High bilirubin levels probably reflect liver congestion secondary to cardiac decompensation in these patients. Further studies are needed to reveal the exact mechanism of this relationship.

This study has some limitations, primarily due to its retrospective design. Second, although all patients were managed with the same protocol in the same clinic, the difference in individual patient-specific strategies may have been reflected in the results. Another deficiency is that preoperative medications are not suitable for presentation due to missing data and a wide range of drug use patterns. Unfortunately, echocardiographic measurements (left atrial volume, etc.) were also not suitable for analysis due to non-standardized reports. Finally, the development of POAF involves multifactorial interactions of different pathophysiologic mechanisms. The distribution of patient characteristics and overlapping comorbidities may have lead to some predictors being underestimated. In addition, stepwise regression may have caused bias in variable selectionNevertheless, we believe in our study reflects the current scenario of daily surgical practice.

## Conclusion

Certain parameters assessed during preoperative physical and laboratory examinations has the potential to be used as markers of POAF, with the advantage of incurring no additional costs in patients scheduled for cardiac surgery. However, since no single pathogenetic mechanism seems to be solely responsible for the development of POAF, a comprehensive set of biochemical and hematologic parameters need to be assessed.

## Data Availability

The datasets used and/or analysed during the current study are available from the corresponding author on reasonable request.

## Abbreviations

AF: Atrial fibrillation
CABG: Coronary artery bypass grafting
COPD: Chronic obstructive pulmonary disease
CPB: Cardiopulmonary bypass
ECG: Electrocardiography
EF: Ejection fraction
GFR: Glomerular filtration rate
OR: Odds ratio
POAF: Postoperative atrial fibrillation

## References

[1] Axtell AL, Moonsamy P, Melnitchouk S, Tolis G, Jassar AS, D'Alessandro DA (2020). Preoperative predictors of new-onset prolonged atrial fibrillation after surgical aortic valve replacement. J Thorac Cardiovasc Surg.

[2] Weymann A, Popov AF, Sabashnikov A, Ali-Hasan-Al-Saegh S, Ryazanov M, Tse G (2018). Baseline and postoperative levels of C-reactive protein and interleukins as inflammatory predictors of atrial fibrillation following cardiac surgery: a systematic review and meta-analysis. Kardiol Pol.

[3] Tadic M, Ivanovic B, Zivkovic N (2011). Predictors of atrial fibrillation following coronary artery bypass surgery. Med Sci Monit.

[4] Li T, Sun ZL, Xie QY (2016). Meta-analysis identifies serum C-reactive protein as an indicator of atrial fibrillation risk after coronary artery bypass graft. Am J Ther.

[5] Bessissow A, Khan J, Devereaux PJ, Alvarez-Garcia J, Alonso-Coello P (2015). Postoperative atrial fibrillation in non-cardiac and cardiac surgery: an overview. J Thromb Haemost.

[6] Nair SG (2010). Atrial fibrillation after cardiac surgery. Ann Card Anaesth.

[7] Danelich IM, Lose JM, Wright SS, Asirvatham SJ, Ballinger BA, Larson D (2014). Practical management of postoperative atrial fibrillation after noncardiac surgery. J Am Coll Surg.

[8] Dobrev D, Aguilar M, Heijman J, Guichard JB, Nattel S (2019). Postoperative atrial fibrillation: mechanisms, manifestations and management. Nat Rev Cardiol.

[9] Turagam MK, Mirza M, Werner PH, Sra J, Kress DC, Tajik AJ (2016). Circulating biomarkers predictive of postoperative atrial fibrillation. Cardiol Rev.

[10] Guan B, Li X, Xue W, Tse G, Waleed KB, Liu Y (2020). Blood lipid profiles and risk of atrial fibrillation: a systematic review and meta-analysis of cohort studies. J Clin Lipidol.

[11] Li XY, Hou HT, Chen HX, Liu XC, Wang J, Yang Q, He GW (2020). Preoperative plasma biomarkers associated with atrial fibrillation after coronary artery bypass surgery. J Thorac Cardiovasc Surg..

[12] Gorczyca I, Michta K, Pietrzyk E, Wożakowska-Kapłon B (2018). Predictors of post-operative atrial fibrillation in patients undergoing isolated coronary artery bypass grafting. Kardiol Pol.

[13] Cerit L, Kemal H, Gulsen K, Ozcem B, Cerit Z, Duygu H (2017). Relationship between Vitamin D and the development of atrial fibrillation after on-pump coronary artery bypass graft surgery. Cardiovasc J Afr.

[14] Chua SK, Shyu KG, Lu MJ, Hung HF, Cheng JJ, Lee SH (2013). Association between renal function, diastolic dysfunction, and postoperative atrial fibrillation following cardiac surgery. Circ J.

[15] January CT, Wann LS, Alpert JS, Calkins H, Cigarroa JE, Cleveland JC (2014). (2014) ACC/AHA Task Force Members: 2014 AHA/ACC/HRS guideline for the management of patients with atrial fibrillation: a report of the American College of Cardiology/American Heart Association Task Force on practice guidelines and the Heart Rhythm Society. Circulation.

[16] Magne J, Salerno B, Mohty D, Serena C, Rolle F, Piccardo A (2019). Echocardiography is useful to predict postoperative atrial fibrillation in patients undergoing isolated coronary bypass surgery: a prospective study. Eur Heart J Acute Cardiovasc Care.

[17] Elawady MA, Bashandy M (2014). Clinical and echocardiographic predicators of postoperative atrial fibrillation. Asian Cardiovasc Thorac Ann.

[18] Zangrillo A, Landoni G, Sparicio D, Benussi S, Aletti G, Pappalardo F (2004). Predictors of atrial fibrillation after off-pump coronary artery bypass graft surgery. J Cardiothorac Vasc Anesth.

[19] Mariscalco G, Biancari F, Zanobini M, Cottini M, Piffaretti G, Saccocci M (2014). Bedside tool for predicting the risk of postoperative atrial fibrillation after cardiac surgery: the POAF score. J Am Heart Assoc.

[20] Conti VR, Ware DL (2002). Cardiac arrhythmias in cardiothoracic surgery. Chest Surg Clin N Am.

[21] Kinoshita T, Asai T, Takashima N, Hosoba S, Suzuki T, Kambara A (2011). Preoperative C-reactive protein and atrial fibrillation after off-pump coronary bypass surgery. Eur J Cardiothorac Surg.

[22] Bruins P, TeVelthuis H, Yazdanbakhsh AP, Jansen PG, vanHardevelt FW, deBeaumont EM (1997). Activation of the complement system during and after cardiopulmonary bypass surgery: postsurgery activation involves C-reactive protein and is associated with postoperative arrhythmia. Circulation.

[23] Şaşkın H, Düzyol Ç, Aksoy R, Özcan KS, Güngör B, İdiz M (2016). Do preoperative C-reactive protein and mean platelet volume levels predict development of postoperative atrial fibrillation in patients undergoing isolated coronary artery bypass grafting?. Postepy Kardiol Interwencyjnej.

[24] Weymann A, Ali-Hasan-Al-Saegh S, Sabashnikov A, Popov AF, Mirhosseini SJ, Liu T (2017). Prediction of new-onset and recurrent atrial fibrillation by complete blood count tests: a comprehensive systematic review with meta-analysis. Med Sci Monit Basic Res.

[25] Weymann A, Ali-Hasan-Al-Saegh S, Popov AF, Sabashnikov A, Mirhosseini SJ, Liu T (2018). Haematological indices as predictors of atrial fibrillation following isolated coronary artery bypass grafting, valvular surgery, or combined procedures: a systematic review with meta-analysis. Kardiol Pol.

[26] Kodani E, Inoue H, Atarashi H, Okumura K, Yamashita T, Origasa H (2020). Impact of hemoglobin concentration and platelet count on outcomes of patients with non-valvular atrial fibrillation: a subanalysis of the J-RHYTHM Registry. Int J Cardiol.

[27] Alameddine AK, Visintainer P, Alimov VK, Rousou JA (2014). Blood transfusion and the risk of atrial fibrillation after cardiac surgery. J Card Surg.

[28] Abbaszadeh S, Shafiee A, Bina P, Jalali A, Sadeghian S, Karimi A (2017). Preoperative hemoglobin A1c and the occurrence of atrial fibrillation following on-pump coronary artery bypass surgery in type-2 diabetic patients. Crit Pathw Cardiol.

[29] Kinoshita T, Asai T, Suzuki T, Kambara A, Matsubayashi K (2012). Preoperative hemoglobin A1c predicts atrial fibrillation after off-pump coronary bypass surgery. Eur J Cardiothorac Surg.

[30] Zhao H, Liu M, Chen Z, Mei K, Yu P, Xie L (2020). Dose-response analysis between hemoglobin A1c and risk of atrial fibrillation in patients with and without known diabetes. PLoS ONE.

[31] Mora S, Akinkuolie AO, Sandhu RK, Conen D, Albert CM (2014). Paradoxical association of lipoprotein measures with incident atrial fibrillation. Circ Arrhythm Electrophysiol.

[32] Lopez FL, Agarwal SK, Maclehose RF, Soliman EZ, Sharrett AR, Huxley R (2012). Blood lipid levels, lipid-lowering medications, and the incidence of atrial fibrillation: the atherosclerosis risk in communities study. Circ Arrhythm Electrophysiol..

[33] Yao Y, Liu F, Wang Y, Liu Z (2020). Lipid levels and risk of new-onset atrial fibrillation: a systematic review and dose-response meta-analysis. Clin Cardiol.

[34] Li ZZ, Du X, Guo XY, Tang RB, Jiang C, Liu NC (2018). Association between blood lipid profiles and atrial fibrillation: a case-control study. Med Sci Monit..

[35] Aydin M, Susam I, Kilicaslan B, Dereli M, Sacar M, Ozdogan O (2014). Serum cholesterol levels and postoperative atrial fibrillation. J Cardiothorac Surg.

[36] Patti G, Bennett R, Seshasai SR, Cannon CP, Cavallari I, Chello M (2015). Statin pretreatment and risk of in-hospital atrial fibrillation among patients undergoing cardiac surgery: a collaborative meta-analysis of 11 randomized controlled trials. Europace.

[37] Yin L, Wang Z, Wang Y, Ji G, Xu Z (2010). Effect of statins in preventing postoperative atrial fibrillation following cardiac surgery. Heart Lung Circ.

[38] Demir M, Demir C, Uyan U, Melek M (2013). The relationship between serum bilirubin concentration and atrial fibrillation. Cardiol Res.

[39] Sun D, Li W, Zheng W, Tan J, Zhang G (2019). Direct bilirubin level is an independent risk factor for atrial fibrillation in thyrotoxic patients receiving radioactive iodine therapy. Nucl Med Commun.

[40] Lin SP, Lin PY, Jiang HL, Long YM, Chen XH (2015). Is serum total bilirubin useful to differentiate cardioembolic stroke from other stroke subtypes?. Neurol Res.

[41] Chen SC, Chung FP, Chao TF, Hu YF, Lin YJ (2019). A link between bilirubin levels and atrial fibrillation recurrence after catheter ablation. J Chin Med Assoc.

